# Immune checkpoint inhibitors for first‐line treatment of advanced non‐small‐cell lung cancer: A systematic review and network meta‐analysis

**DOI:** 10.1111/1759-7714.14148

**Published:** 2021-09-20

**Authors:** Tzu‐Rong Peng, Hung‐Hong Lin, Fang‐Pei Tsai, Ta‐Wei Wu

**Affiliations:** ^1^ Department of Pharmacy Taipei Tzu Chi Hospital, Buddhist Tzu Chi Medical Foundation New Taipei City Taiwan; ^2^ Department of Pharmacy Chia‐Nan University of Pharmacy and Science Tainan Taiwan; ^3^ School of Pharmacy, College of Pharmacy Taipei Medical University Taipei City Taiwan

**Keywords:** immune checkpoint inhibitors, non‐small cell lung cancer, overall survival, progression‐free survival

## Abstract

**Objective:**

Currently, several immune checkpoint inhibitors (ICIs) treatment for advanced non‐small‐cell lung cancer (NSCLC) have been investigated; their overall efficacy and safety remain unclear.

**Methods:**

We searched electronic databases such as PubMed, EMBASE, and the Cochrane library. The randomized controlled trials (RCTs) that compared ICIs with or without chemotherapy to chemotherapy in advanced NSCLC. We collected and compaired thier parameters, including overall survival (OS), progression‐free survival (PFS), objective response rate (ORR), and treatment‐related adverse events (TRAEs) of grade ≥3.

**Results:**

A total of 15 RCTs involving 8869 patients with NSCLC were included. Pembrolizumab plus platinum‐based chemotherapy had higher OS and PFS than platinum‐based chemotherapy (hazard ratio [HR] 0.55, 95% CI 0.46–0.67; HR 0.54, 95% CI 0.41–0.70, respectively). Pembrolizumab plus platinum‐based chemotherapy had higher ranked ORR than platinum‐based chemotherapy (odds ratio [OR] 2.92, 95% CI 1.99–4.22). In terms of OS, atezolizumab, pembrolizumab plus platinum‐based chemotherapy, and nivolumab plus ipilimumab ranked as the best treatments for patients with programmed death‐ligand 1 (PD‐L1) expression levels of ≥50%, 1–49%, and <1%, respectively. In terms of PFS, pembrolizumab plus platinum‐based chemotherapy ranked as the best treatment for patients with any PD‐L1 expression levels. However, ipilimumab plus platinum‐based chemotherapy, nivolumab plus platinum‐based chemotherapy, and atezolizumab plus platinum‐based chemotherapy have higher TRAEs of grade ≥3 than platinum‐based chemotherapy.

**Conclusions:**

Pembrolizumab plus platinum‐based chemotherapy prevailed in rank in OS, PFS, and ORR benefit. The TRAEs of pembrolizumab plus platinum‐based chemotherapy were more than ICI monotherapy and chemotherapy.

## Graphical abstract

1. Clustered ranking plot for progression‐free survival and grade 3–5 adverse events. Cluster techniques (single linkage clustering) were used to cluster interventions in groups defined by different colors.
2. We use different colors to cluster drugs into groups. It seems that pembrolizumab plus platinum‐based chemotherapy is the most effective but has moderate grade 3–5 adverse events).

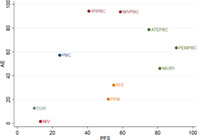

## INTRODUCTION

Lung cancer is the leading cause of cancer‐related deaths worldwide, and most patients first have lung cancer diagnosed as an advanced stage with metastasis.^1^ The 5‐year survival rate is only 16%.^2^ Non‐small‐cell lung cancer (NSCLC) manifests as the most common histological subtype of lung cancer.^3^ Around 70% of patients with lung cancer are first diagnosed with an advanced or metastatic stage of lung cancer.^4^ There are some patients with locally advanced or metastatic lung cancer, which cannot be surgically removed. Thus, platinum‐based chemotherapy of docetaxel and/or radiotherapy is often the first choice for treatment.^5^ However, even with these therapies, most patients still cannot obtain an effective prognosis.^6^ Therefore, in recent years, antitumor for the immune system, named immunotherapy, will become one of the treatment options.^7^

Programmed death 1 (PD‐1), programmed death‐ligand 1 (PD‐L1), and T‐cell lymphocyte antigen 4 (CTLA‐4) inhibitors have shown clinical activity and marked efficacy in the treatment of NSCLC. The efficacy of immune checkpoint inhibitors (ICIs) in the treatment of advanced NSCLC is obvious, with a 3‐year overall survival (OS) rate of 19% in previously treated patients and 26.4% in treatment‐naïve patients, and more than 18 months of progression‐free survival (PFS).^8^ The efficacy and safety of ICIs for patients with advanced NSCLC remain controversial. There are several regimens of ICIs, including monotherapy ICIs (avelumab [AVE], atezolizumab [ATE], durvalumab [DUR], ipilimumab [IPI], nivolumab [NIV], pembrolizumab [PEM]) and ICI combination with chemotherapy (platinum‐based chemotherapy [PBC]). ICI monotherapy or ICIs plus chemotherapy have confirmed an alternative option of first‐ or second‐line treatment for patients with advanced NSCLC.^9^, ^10^ Moreover, the most important issue is that no prospective head‐to‐head randomized control trials have compared the efficacy and safety of PD‐1, PD‐L1, and CTLA‐4 inhibitors. Therefore, we conducted a network meta‐analysis to investigate the best choice of ICIs for first‐line treatment of advanced NSCLC.

## MATERIALS AND METHODS

### Search strategy and study selection

We performed a network meta‐analysis by searching PubMed, the Cochrane Library, and EMBASE for relevant literature published up to 31 July 2021. The following search terms were used: ICIs (anti‐PD‐1 or anti‐PD‐L1 or anti‐CTLA‐4 or programmed death 1 or PD‐1 or programmed death‐ligand 1 or PD‐L1 or immunotherapy or immune checkpoint inhibitors or PD‐1/PD‐L1 inhibitors or PD‐1/PD‐L1 blockade or anti‐PD‐1/PD‐L1), specific ICI drug names (avelumab, atezolizumab, durvalumab, ipilimumab, nivolumab, pembrolizumab, tremelimumab), and lung cancer (non‐small‐cell lung cancer or non‐small cell lung carcinoma or non‐small cell lung neoplasms or lung adenocarcinoma or lung squamous cell carcinoma). Eligible studies were RCTs and reported on OS, PFS, ORR, and adverse events. All retrieved abstracts, studies, and citations were reviewed. Additionally, we searched the reference sections of the selected papers for relevant studies. The search was limited to English articles and those that involved humans. The detailed information on the search strategy for eligible studies is given in the flowchart provided by Preferred Reporting Items for Systematic Reviews and Meta‐Analyses (PRISMA).^11^ The retrieved studies were independently reviewed by two reviewers (T.‐R.P. and T.‐W.W.). Any discrepancies between the reviewers were resolved by consensus (F.‐P.T.).

### Data collection, inclusion criteria, and excluded criteria

This study was performed following Cochrane Collaboration guidelines.^12^ The following information was extracted: trial ID, first author, publication year, study design, phase of the trial, histology type, number of enrolled patients, OS, PFS, ORR, and TRAEs of grade ≥3. Trials that met the following criteria were included: (1) randomized control trial, (2) advanced‐stage NSCLC, (3) treated with PD‐1, PD‐L1, CTLA‐4 inhibitors (avelumab, atezolizumab, durvalumab, ipilimumab, nivolumab, pembrolizumab, tremelimumab) with or without chemotherapy, (4) comparison treated with chemotherapy, and (5) outcomes OS and PFS measured as hazard ratios (HRs), ORR measured as odds ratios (ORs), and treatment‐related adverse events (TRAEs) of grade≥3 measured as risk ratios (RR). No restriction in the publication year of the studies was implemented. Studies were excluded based on the following criteria as follows: (1) non‐RCT studies such as retrospective, prospective observational cohort studies or reviews, case reports, letters, commentaries, editorials, or meta‐analysis, (2) lack of related data, and (3) non‐first‐line treatment with PD‐1, PD‐L1, and CTLA‐4 inhibitors.

### Methodological quality appraisal

Two reviewers (T.‐R.P. and T.‐W.W.) independently assessed the methodological quality of each study by using the revised risk‐of‐bias (version 2.0) method, according to the recommendation of the Cochrane Collaboration.^13^ Several domains were assessed, including the adequacy of randomization, allocation concealment, blinding of patients and outcome assessors, length of follow‐up, the information provided to patients regarding study withdrawal, whether intention‐to‐treat analysis was performed, and freedom from other biases.

### Statistical analyses

This network meta‐analysis applied the frequentist approach model. Statistical evaluation of inconsistency and production of network graphs and figures were performed using the network and network graphs packages in STATA version 15 (STATA Corporation). A network meta‐analysis was performed by using hazard ratios for survival outcomes (progression‐free survival and overall survival), odds ratios for objective response rate, and risk ratios for binary outcomes (grade ≥3 adverse events) along with corresponding 95% confidence intervals for indirect and mixed comparisons. We tested for possible inconsistency globally using a *χ* ^2^‐test, and locally by calculating inconsistency factors for each comparison in closed loops. We estimated the ranking probabilities of being at each possible rank for each intervention. We used comparison‐adjusted funnel plots to assess publication bias.

## RESULTS

### Literature search results

**FIGURE 1:**
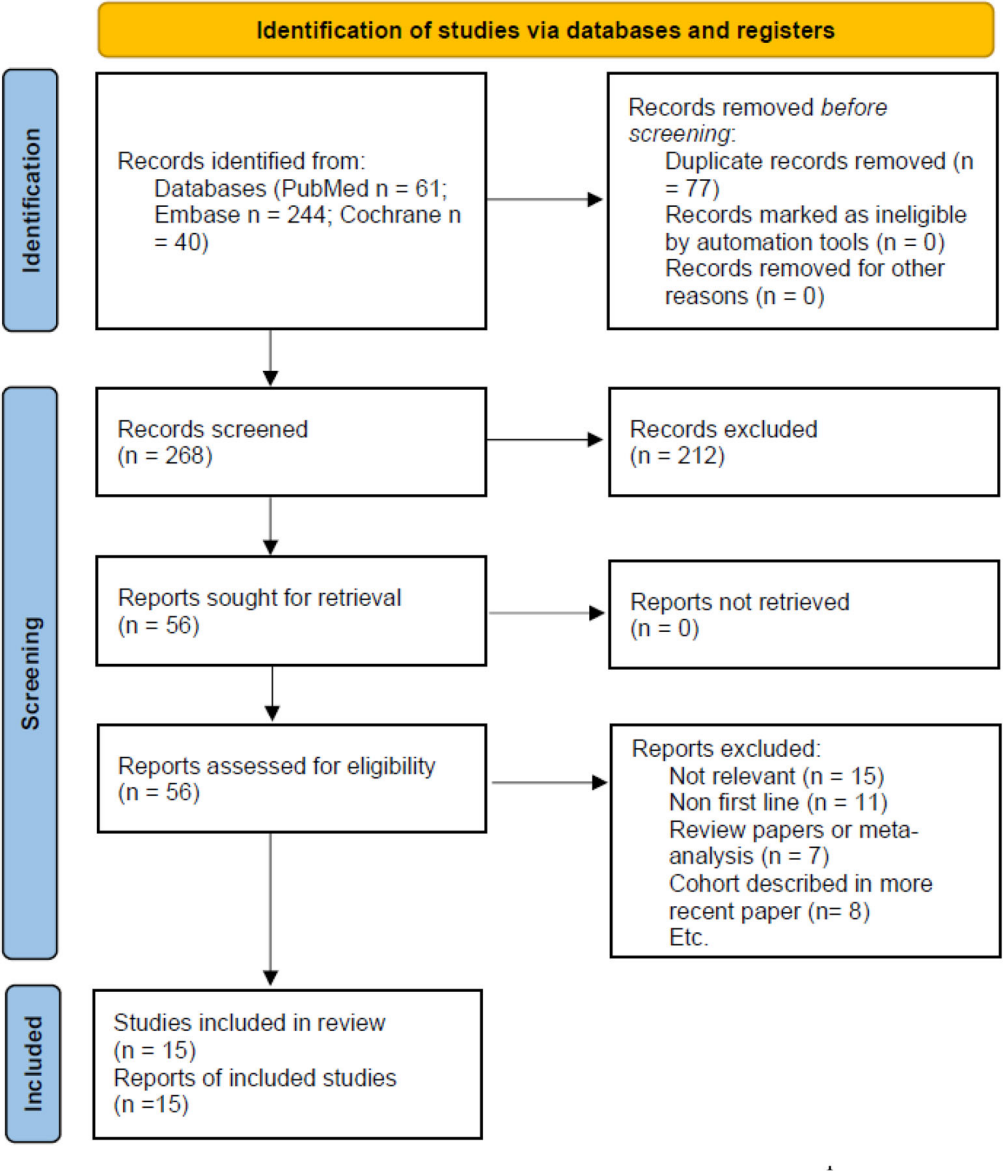
Flow diagram of the studies identified^11^

We identified 345 records from the PubMed, EMBASE, and Cochrane electronic databases. Seventy‐seven studies were removed due to duplication. After the exclusion of duplication studies, we reviewed 268 studies based on title and abstract, and 212 studies were removed because of irrelevant records. Of the 56 studies that underwent the review of a full article, 41 were removed. Finally, 15 studies matched our inclusion criteria. The PRISMA flowchart shows the detailed process of study selection (Figure 1).

### Eligible studies and patient characteristics

The basic characteristics of the eligible studies and patients are presented in Table 1. The extracted outcome data with PD‐L1 expression from all included studies are shown in Table 2. All selected studies were RCTs published between 2016 and 2020. All studies were phase III clinical trials. A total of 8869 patients were included in the analysis (4651 for the PD‐1, PD‐L1, CTLA‐4 inhibitors group, and 4218 for the chemotherapy group). All 15 studies had two arm interventions. The risk of bias assessment is shown in Supporting Information Figure S1.

**TABLE 1:** The baseline characteristics of selected studies

Author	Year	Disease stage	Study phase	No. of patients	Treatment	OS (95% CI)	PFS (95% CI)	ORR	Grade 3–5 TRAEs
Reck et al.^14^	2016	IV	III	154	Pembrolizumab	0.60 (0.41–0.89)	0.50 (0.37–0.68)	69 (154)	41 (113)
				151	Platinum‐based chemotherapy			42 (151)	80 (150)
Langer et al.^15^	2016	IIB	III	60	Pembrolizumab plus platinum‐based chemotherapy	0.56 (0.32–0.95)	0.53 (0.33–0.86)	33 (60)	24 (59)
		IV		63	Platinum‐based chemotherapy			18 (63)	17 (62)
Govindan et al.^16^	2017	IV	III	388	Ipilimumab plus platinum‐based chemotherapy	0.91 (0.77–1.07)	0.87 (0.75–1.01)	171 (388)	205 (388)
		Recurrent		361	Platinum‐based chemotherapy			170 (361)	129 (361)
Carbone et al.^17^	2017	IV	III	271	Nivolumab	1.08 (0.87–1.34)	1.19 (0.97–1.46)	55 (211)	47 (267)
		Recurrent		270	Platinum‐based chemotherapy			71 (212)	133 (263)
Jotte et al.^18^	2018	IV	III	343	Atezolizumab plus platinum‐based chemotherapy	0.96 (0.78–1.18)	0.71 (0.60–0.85)	169 (343)	231 (334)
		Recurrent		340	Platinum‐based chemotherapy			140 (340)	193 (334)
Papadimitrakopoulou et al.^19^	2018	IV	III	292	Atezolizumab plus platinum‐based chemotherapy	0.81 (0.64–1.03)	0.60 (0.49–0.72)	137 (292)	167 (291)
				286	Platinum‐based chemotherapy			92 (286)	114 (274)
Socinski et al.^20^	2018	IV	III	400	Atezolizumab plus platinum‐based chemotherapy	0.78 (0.64–0.96)	0.62 (0.52–0.74)	224 (353)	230 (393)
		Recurrent		400	Platinum‐based chemotherapy			159 (331)	197 (394)
Gandhi et al.^21^	2018	IV	III	410	Pembrolizumab plus platinum‐based chemotherapy	0.49 (0.38–0.64)	0.52 (0.43–0.64)	195 (410)	272 (405)
				206	Platinum‐based chemotherapy			39 (206)	133 (202)
Paz‐Ares et al.^22^	2018	IV	III	278	Pembrolizumab plus platinum‐based chemotherapy	0.64 (0.49–0.85)	0.56 (0.45–0.70)	161 (278)	194 (278)
				281	Platinum‐based chemotherapy			108 (281)	191 (280)
Hellmann^23^	2018	IV	III	139	Nivolumab plus Ipilimumab	NR	0.58 (0.41–0.81)	63 (139)	180 (576)
		Recurrent		160	Platinum‐based chemotherapy			43 (160)	206 (570)
Borghaei^24^	2018	IV	III	177	Nivolumab plus platinum‐based chemotherapy	NR	0.74 (0.58–0.94)	65 (177)	89 (172)
		Recurrent		186	Platinum‐based chemotherapy			43 (186)	64 (183)
West et al.^25^	2019	IV	III	451	Atezolizumab plus platinum‐based chemotherapy	0.79 (0.64–0.98)	0.64 (0.54–0.77)	220 (447)	354 (473)
				228	Platinum‐based chemotherapy			72 (226)	141 (232)
MOK et al.^26^	2019	IIIB, IV	III	637	Pembrolizumab	0.81 (0.71–0.93)	1.07 (0.94–1.21)	174 (637)	113 (636)
				637	Platinum‐based chemotherapy			169 (637)	252 (615)
Rizvi et al.^27^	2020	IV	III	374	Durvalumab	0.96 (0.81–1.13)	1.24 (1.04–1.48)	NR	55 (369)
				372	Platinum‐based chemotherapy			NR	119 (352)
Herbst et al.^28^	2020	IV	III	277	Atezolizumab	0.83 (0.65–1.07)	0.77 (0.63–0.94)	NR	97 (286)
				277	Platinum‐based chemotherapy			NR	149 (263)

*Abbreviations*: CI, confidence interval; NR; ORR, objective response rate; OS, overall survival; PFS, progression‐free survival; TRAE, treatment‐related adverse events; NR, not reported.

**TABLE 2:** Extracted outcome data with PD‐L1 expression from all included studies

Author	OS‐HR (95% CI)			PFS‐HR (95% CI)		
	PD‐L1 ≥ 50%	PD‐L1 1–49%	PD‐L1 < 1%	PD‐L1 ≥ 50%	PD‐L1 1–49%	PD‐L1 < 1%
Reck et al.^14^	0.62 (0.48–0.81)	NR	NR	0.50 (0.37–0.68)	NR	NR
Langer et al.^15^	NR	NR	NR	NR	NR	NR
Govindan et al.^16^	NR	NR	NR	NR	NR	NR
Carbone et al.^17^	0.90 (0.63–1.29)	NR	NR	1.07 (0.77–1.49)	NR	NR
Jotte et al.^18^	0.48 (0.29–0.81)	1.08 (0.81–1.45)	0.87 (0.67–1.13)	0.41 (0.25–0.68)	0.70 (0.54–0.91)	0.82 (0.65–1.04)
Papadimitrakopoulou et al.^19^	0.73 (0.31–1.73)	1.18 (0.80–1.76)	0.67 (0.46–0.96)	0.46 (0.22–0.96)	0.80 (0.55–1.16)	0.45 (0.31–0.64)
Socinski et al.^20^	NR	NR	NR	0.39 (0.25–0.60)	0.50 (0.39–0.64)	0.77 (0.61–0.99)
Gandhi et al.^21^	0.59 (0.39–0.88)	0.62 (0.42–0.92)	0.52 (0.36–0.74)	0.36 (0.26–0.51)	0.51 (0.36–0.73)	0.64 (0.47–0.89)
Paz‐Ares et al.^22^	0.79 (0.52–1.21)	0.59 (0.42–0.84)	0.79 (0.56–1.11	0.43 (0.29–0.63)	0.52 (0.38–0.71)	0.67 (0.49–0.91
West et al.^25^	0.84 (0.51–1.39)	0.70 (0.45–1.08)	0.81 (0.61–1.08)	0.51 (0.34–0.77)	0.61 (0.43–0.85)	0.72 (0.56–0.91)
MOK et al.^26^	0.70 (0.58–0.86)	0.91 (0.77–1.09)	NR	0.83 (0.69–1.00)	1.27 (1.08–1.50)	NR
Paz‐Ares et al.^29^	NR	NR	NR	NR	NR	NR
Ramalingam et al.^30^	0.70 (0.55–0.90)	NR	0.62 (0.48–0.78)	0.62 (0.49–0.79)	NR	0.75 (0.59–0.96)
Rizvi et al.^27^	0.76 (0.55–1.04)	NR	1.18 (0.86–1.62)	NR	NR	NR
Herbst et al.^28^	0.59 (0.40–0.89)	1.04 (0.76–1.44)	NR	0.63 (0.45–0.88)	0.90 (0.71–1.15)	NR

*Abbreviations*: CI, confidence interval; HR, hazard ratio; NR; OS, overall survival; PD‐L1, programmed death‐ligand 1; PFS, progression‐free survival; NR, not reported.

### Network geometry and testing for inconsistency

**FIGURE 2:**
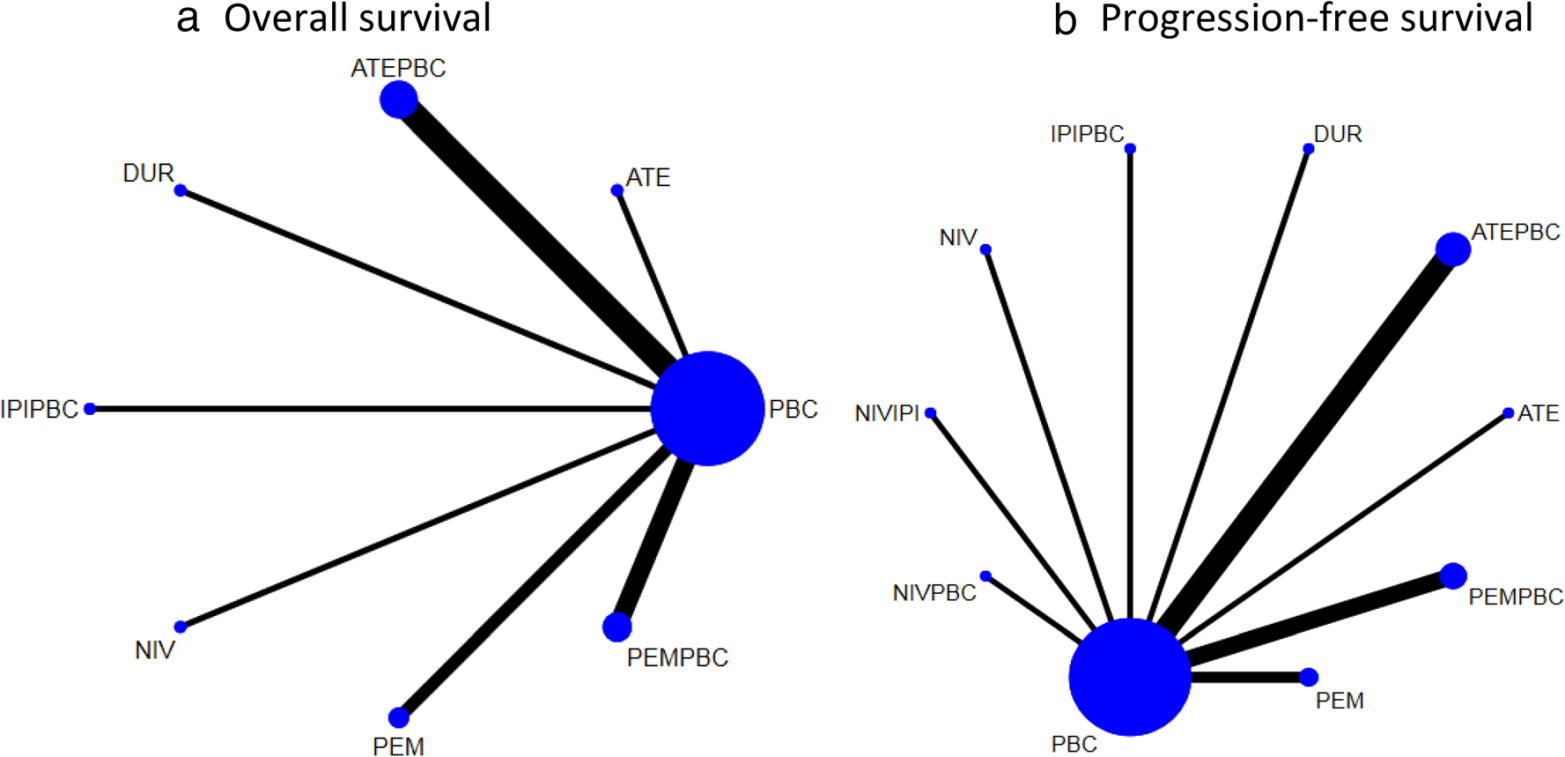
Network construction for comparison in (a) overall survival and (b) progression‐free survival. ATE, atezolizumab; ATEPBC, atezolizumab and platinum‐based chemotherapy; DUR, durvalumab; IPIPBC, ipilimumab and platinum‐based chemotherapy; NIV, nivolumab; NIVIPI, nivolumab and ipilimumab; NIVPBC, nivolumab and platinum‐based chemotherapy; PEM, pembrolizumab; PEMPBC, pembrolizumab and platinum‐based chemotherapy; PBC, platinum‐based chemotherapy

The network constructions are presented in Figure 2. For OS, PFS, ORR, and grade ≥3 adverse events, five ICIs plus chemotherapy or without chemotherapy and chemotherapy alone were included in the network meta‐analysis. A test for inconsistency was not done since the evidence network did not have a combination of direct and indirect evidence.

### Efficacy evaluation from the network meta‐analysis

**FIGURE 3:**
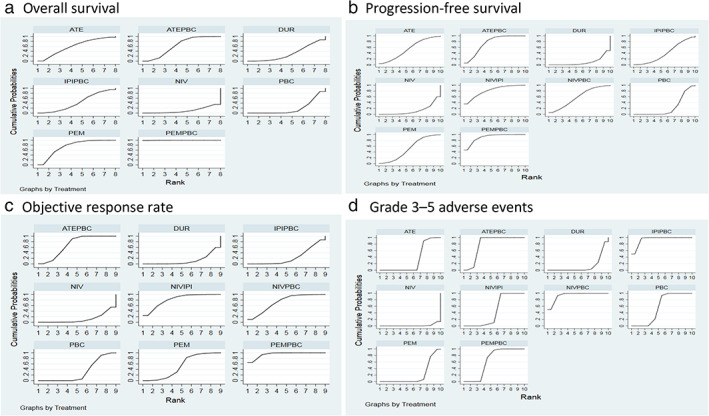
Cumulative ranking probability for different treatments: (a) overall survival, (b) progression‐free survival, (c) objective response rate, and (d) grade 3–5 adverse events. ATE, atezolizumab; ATEPBC, atezolizumab and platinum‐based chemotherapy; DUR, durvalumab; IPIPBC, ipilimumab and platinum‐based chemotherapy; NIV, nivolumab; NIVIPI, nivolumab and ipilimumab; NIVPBC, nivolumab and platinum‐based chemotherapy; PEM, pembrolizumab; PEMPBC, pembrolizumab and platinum‐based chemotherapy; PBC, platinum‐based chemotherapy

Regarding OS, three drugs (pembrolizumab plus platinum‐based chemotherapy, pembrolizumab, and atezolizumab plus platinum‐based chemotherapy) showed a significant improvement on OS compared to platinum‐based chemotherapy (Table 3). There was a significant difference in OS across the two highest‐ranking drugs (HR 0.72, 95% CI 0.55, 0.93). Indirect comparisons of drugs superior to platinum‐based chemotherapy showed greater surface under the cumulative ranking curve (SUCRA) values for pembrolizumab plus platinum‐based chemotherapy (1.0), pembrolizumab (0.75), atezolizumab plus platinum‐based chemotherapy (0.63), atezolizumab (0.60), ipilimumab plus platinum‐based chemotherapy (0.42), durvalumab (0.31), platinum‐based chemotherapy (0.19), and nivolumab (0.1) (Figure 3a). Pembrolizumab plus platinum‐based chemotherapy had the highest probability (98.5%) of ranking as the best treatment. Pembrolizumab had the highest probability (53.2%) of ranking as the second‐best treatment (Table 7A). Regarding PFS, three drugs (pembrolizumab plus platinum‐based chemotherapy, nivolumab plus ipilimumab, and atezolizumab plus platinum‐based chemotherapy) showed a significant improvement on PFS compared to platinum‐based chemotherapy (Table 4). There was no significant difference in PFS across the three highest‐ranking drugs. The SUCRA ranking suggested pembrolizumab plus platinum‐based chemotherapy (0.91) as the best intervention followed by nivolumab plus ipilimumab (0.81), atezolizumab plus platinum‐based chemotherapy (0.75), nivolumab plus platinum‐based chemotherapy (0.59), atezolizumab (0.55), pembrolizumab (0.52), ipilimumab plus platinum‐based chemotherapy (0.41), platinum‐based chemotherapy (0.24), nivolumab (0.13), and durvalumab (0.1) (Figure 3b). Pembrolizumab plus platinum‐based chemotherapy had the highest probability (47.0%) of ranking as the best treatment. Pembrolizumab plus platinum‐based chemotherapy had the highest probability (32.4%) of ranking as the second‐best treatment (Table 7B).

**TABLE 3:** Network meta‐analysis of overall survival, presented as constant hazard ratios between all competing interventions with 95% confidence intervals

PEMPBC							
**0.72** (0.55,0.93)	**PEM**						
**0.72** (0.55,0.93)	0.93 (0.76,1.15)	**ATEPBC**					
**0.67** (0.49,0.93)	0.93 (0.68,1.28)	1 (0.75,1.34)	**ATE**				
**0.61** (0.47,0.79)	0.85 (0.66,1.11)	0.91 (0.73,1.14)	0.91 (0.66,1.26)	**IPIPBC**			
**0.58** (0.44,0.76)	0.81 (0.63,1.04)	0.87 (0.7,1.08)	0.86 (0.63,1.2)	0.95 (0.73,1.23)	**DUR**		
**0.55** (0.46,0.67)	**0.78** (0.65,0.92)	**0.84** (0.74,0.93)	0.83 (0.64,1.08)	0.91 (0.76,1.09)	0.96 (0.79,1.16)	**PBC**	
**0.52** (0.38,0.7)	**0.78** (0.65,0.92)	**0.77** (0.59,1)	0.77 (0.54,1.09)	0.84 (0.63,1.14)	0.89 (0.66,1.2)	0.92 (0.73,1.17)	**NIV**

*Note*: Boldface indicate statistical significance.

*Abbreviations*: ATE, atezolizumab; ATEPBC, atezolizumab and platinum‐based chemotherapy; DUR, durvalumab; IPIPBC, ipilimumab and platinum‐based chemotherapy; NIV, nivolumab; PEM, pembrolizumab; PEMPBC, pembrolizumab and platinum‐based chemotherapy; PBC, platinum‐based chemotherapy.

**TABLE 4:** Network meta‐analysis of progression‐free survival, presented as constant hazard ratios between all competing interventions with 95% confidence intervals

PEMPBC									
0.92 (0.52,1.65)	**NIVIPI**								
0.84 (0.59,1.19	0.9 (0.52,1.57)	**ATEPBC**							
0.73 (0.43,1.23)	0.79 (0.4,1.55)	0.87 (0.53,1.42)	**NIVPBC**						
0.7 (0.42,1.16)	0.76 (0.39,1.46)	0.84 (0.52,1.35)	0.96 (0.52,1.79)	**ATE**					
0.68 (0.45,1.04)	0.73 (0.4,1.35)	0.82 (0.55,1.2)	0.94 (0.54,1.63)	0.98 (0.57,1.67)	**PEM**				
0.62 (0.38,1.01)	0.66 (0.35,1.28)	0.74 (0.47,1.16)	0.85 (0.46,1.57)	0.89 (0.49,1.6)	0.9 (0.54,1.52)	**IPIPBC**			
**0.54** (0.41,0.7)	**0.58** (0.35,0.97)	**0.64** (0.52,0.79)	0.74 (0.47,1.16)	0.77 (0.5,1.19)	0.79 (0.57,1.08)	0.87 (0.58,1.31	**PBC**		
**0.45** (0.27,0.76)	**0.49** (0.25,0.95)	**0.54** (0.33,0.87)	0.62 (0.33,1.16)	0.64 (0.35,1.19)	0.66 (0.39,1.13)	0.73 (0.4,1.32)	0.84 (0.55,1.3)	**NIV**	
**0.43** (0.26,0.71)	**0.47** (0.24,0.9)	**0.52** (0.32,0.83)	0.59 (0.32,1.11)	0.62 (0.34,1.13)	0.64 (0.38,1.07)	0.7 (0.39,1.26)	0.8 (0.53,1.22)	0.96 (0.53,1.75)	**DUR**

*Note*: Boldface indicate statistical significance.

*Abbreviations*: ATE, atezolizumab; ATEPBC, atezolizumab and platinum‐based chemotherapy; DUR, durvalumab; IPIPBC, ipilimumab and platinum‐based chemotherapy; NIV, nivolumab; NIVIPI, nivolumab and ipilimumab; NIVPBC, nivolumab and platinum‐based chemotherapy; PEM, pembrolizumab; PEMPBC, pembrolizumab and platinum‐based chemotherapy; PBC, platinum‐based chemotherapy.

When it comes to ORR, four drugs (pembrolizumab plus platinum‐based chemotherapy, nivolumab plus ipilimumab, nivolumab plus platinum‐based chemotherapy, and atezolizumab plus platinum‐based chemotherapy) showed a significant improvement on ORR compared to platinum‐based chemotherapy (Table 5). Indirect comparisons of drugs superior to platinum‐based chemotherapy showed greater surface under cumulative ranking curve values for pembrolizumab plus platinum‐based chemotherapy (0.95), nivolumab plus ipilimumab (0.82), nivolumab plus platinum‐based chemotherapy (0.73), atezolizumab plus platinum‐based chemotherapy (0.69), pembrolizumab (0.52), platinum‐based chemotherapy (0.32), and durvalumab (0.12) than for nivolumab (0.11) (Figure 3c). Pembrolizumab plus platinum‐based chemotherapy had the highest probability (65.0%) of ranking as the best treatment. Nivolumab plus ipilimumab had the highest probability (33.9%) of ranking as the second‐best treatment (Table 7C).

**TABLE 5:** Network meta‐analysis of objective response rate, presented as constant odds ratios between all competing interventions with 95% confidence intervals

PEMPBC								
1.28 (0.61,2.75)	**NIVIPI**							
1.51 (0.72,3.16)	1.17 (0.47,2.94)	**NIVPBC**						
**1.63** (1.03,2.59)	1.27 (0.62,2.59)	1.08 (0.54,2.18)	**ATEPBC**					
**2.14** (1.23,3.71)	1.67 (0.76,3.6)	1.42 (0.66,3.03)	1.31 (0.79,2.14)	**PEM**				
**2.92** (1.99,4.22)	**2.25** (1.16,4.35)	**1.93** (1.02,3.67)	**1.77** (1.35,2.34)	1.36 (0.9,2.05)	**PBC**			
**3.29** (1.72,6.3)	**2.56** (1.09,5.93)	2.18 (0.95,5.0)	**2.01** (1.11,3.67)	1.54 (0.78,3)	1.13 (0.66,1.92)	**IPIPBC**		
**4.14** (2.01,8.5)	**3.22** (1.31,7.92)	**2.75** (1.14,6.69)	**2.53** (1.3,4.95)	1.93 (0.92,4.06)	1.43 (0.77,2.64)	1.26 (0.56,2.86)	**NIV**	
**4.06** (2.1,7.92)	**3.16** (1.34,7.46)	**2.69** (1.16,6.3)	**2.48** (1.34,4.62)	1.9 (0.95,3.82)	1.4 (0.8,2.44)	1.23 (0.58,2.66)	0.98 (0.43,2.25)	**DUR**

*Note*: Boldface indicate statistical significance.

*Abbreviations*: ATEPBC, atezolizumab and platinum‐based chemotherapy; DUR, durvalumab; IPIPBC, ipilimumab and platinum‐based chemotherapy; NIV, nivolumab; NIVIPI, nivolumab and ipilimumab; NIVPBC, nivolumab & platinum‐based chemotherapy; PBC, platinum‐based chemotherapy; PEM, pembrolizumab; PEMPBC, pembrolizumab and platinum‐based chemotherapy.

### Safety evaluation from the network meta‐analysis

**FIGURE 4:**
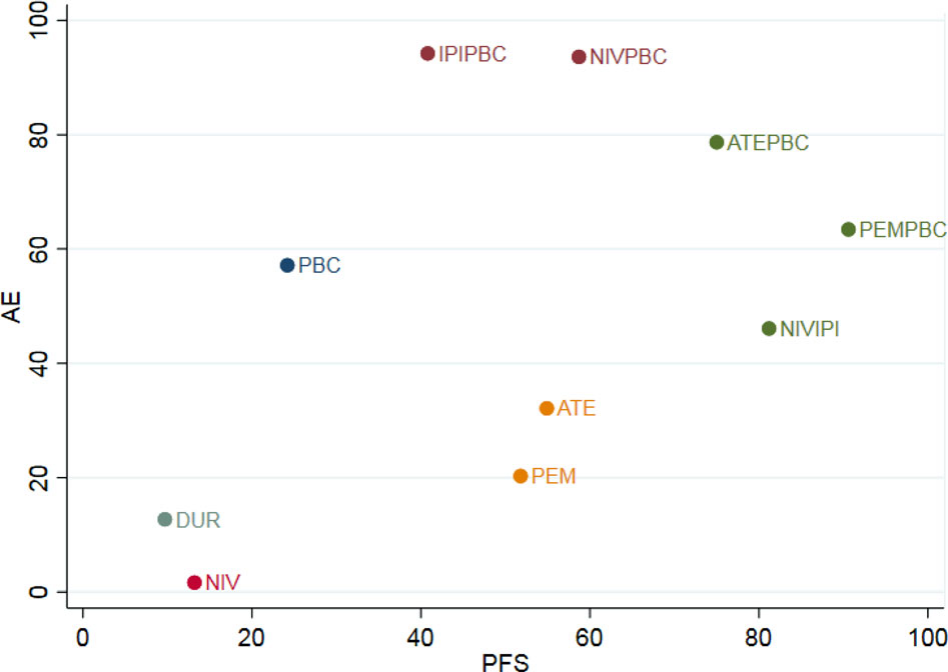
Clustered ranking plot for progression‐free survival and grade 3–5 adverse events. Cluster techniques (single linkage clustering) were used to cluster interventions in groups defined by different colors. ATE, atezolizumab; ATEPBC, atezolizumab and platinum‐based chemotherapy; DUR, durvalumab; IPIPBC, ipilimumab and platinum‐based chemotherapy; NIV, nivolumab; NIVIPI, nivolumab and ipilimumab; NIVPBC, nivolumab and platinum‐based chemotherapy; PEM, pembrolizumab; PEMPBC, pembrolizumab and platinum‐based chemotherapy; PBC, platinum‐based chemotherapy

In terms of treatment‐related adverse events (TRAEs) of grade≥3, three drugs (ipilimumab plus platinum‐based chemotherapy, nivolumab plus platinum‐based chemotherapy, tezolizumab plus platinum‐based chemotherapy) showed significantly greater TRAEs of grade ≥3 compared to platinum‐based chemotherapy (Table 6). The greater TRAEs of grade ≥3 of SUCRA values for ipilimumab plus platinum‐based chemotherapy (0.94), nivolumab plus platinum‐based chemotherapy (0.93), atezolizumab plus platinum‐based chemotherapy (0.79), pembrolizumab plus platinum‐based chemotherapy (0.63), platinum‐based chemotherapy (0.57), nivolumab plus ipilimumab (0.46), atezolizumab (0.32), pembrolizumab (0.20), durvalumab (0.13) than for nivolumab (0.02) (Figure 3d). Nivolumab plus platinum‐based chemotherapy had the highest probability (50.3%) of ranking as the best treatment. Ipilimumab plus platinum‐based chemotherapy had the highest probability (49.1%) of ranking as the second‐best treatment (Table 7D). Figure 4 plots a scatterplot between the SUCRA values for efficacy (progression‐free survival) and tolerability (grade 3–5 adverse events) of treatment drugs. We use different colors to cluster drugs into groups. It seems that pembrolizumab plus platinum‐based chemotherapy is the most effective but has moderate grade 3–5 adverse events).

**TABLE 6:** Network meta‐analysis of treatment‐related adverse events of grade ≥3, presented as constant risk ratios between all competing interventions with 95% confidence intervals

IPIPBC									
1 (0.74,1.35)	**NIVPBC**								
**1.21** (1.01,1.45)	1.21 (0.94,1.55)	**ATEPBC**							
**1.43** (1.19,1.73)	**1.43** (1.11,1.86)	**1.19** (1.07,1.32)	**PEMPBC**						
**1.48** (1.25,1.75)	**1.48** (1.16,1.9)	**1.22** (1.15,1.31)	1.03 (0.95,1.12)	**PBC**					
**1.65** (1.31,2.1)	**1.65** (1.23,2.23)	**1.38** (1.15,1.63)	1.15 (0.96,1.38)	1.12 (0.95,1.31)	**NIVIPI**				
**2.46** (1.92,3.19)	**2.46** (1.8,3.39)	**2.05** (1.67,2.51)	**1.72** (1.39,2.12)	**1.67** (1.38,2.03)	**1.49** (1.16,1.92)	**ATE**			
**2.97** (2.36,3.74)	**2.97** (2.23,3.97)	**2.46** (2.08,2.92)	**2.08** (1.73,2.48)	**2.01** (1.72,2.36)	**1.79** (1.43,2.25)	1.2 (0.93,1.54)	**PEM**		
**3.35** (2.41,4.66)	**3.35** (2.32,4.9)	**2.77** (2.08,3.71)	**2.34** (1.73,3.16)	**2.27** (1.7,3)	**2.03** (1.46,2.8)	1.36 (0.96,1.92)	1.13 (0.82,1.57)	**DUR**	
**4.26** (3.06,5.93)	**4.26** (2.92,6.17)	**3.53** (2.64,4.71)	**2.97** (2.2,3.97)	**2.89** (2.16,3.82)	**2.56** (1.84,3.56)	**1.72** (1.22,2.44)	**1.43** (1.03,1.99)	1.27 (0.84,1.9)	**NIV**

*Note*: Boldface indicate statistical significance.

*Abbreviations*: ATE, atezolizumab; ATEPBC, atezolizumab and platinum‐based chemotherapy; DUR, durvalumab; IPIPBC, ipilimumab and platinum‐based chemotherapy; NIV, nivolumab; NIVIPI, nivolumab and ipilimumab; NIVPBC, nivolumab and platinum‐based chemotherapy; PBC, platinum‐based chemotherapy; PEM, pembrolizumab; PEMPBC, pembrolizumab and platinum‐based chemotherapy.

**TABLE 7:** Rank probability of being the best treatment (PrBest) by (A) overall survival, (B) progression‐free survival, (C) objective response rate, and (D) grade 3–5 adverse events

(A) Overall survival								
Study and rank	PBC	ATE	ATEPBC	DUR	IPIPBC	NIV	PEM	PEMPBC
Best	0.0%	0.8%	0.0%	0.0%	0.0%	0.0%	0.7%	**98.5%**
2nd	0.0%	26.6%	12.9%	1.4%	4.3%	0.2%	**53.2%**	1.4%
3rd	0.0%	22.5%	**34.9%**	4.6%	10.2%	1.2%	26.5%	0.1%
4th	0.3%	19.7%	34.9%	11.0%	18.9%	2.3%	12.8%	0.0%
5th	6.0%	14.0%	13.6%	22.7%	32.0%	6.4%	5.3%	0.0%
6th	31.8%	8.1%	3.1%	26.0%	18.3%	11.3%	1.4%	0.0%
7th	48.8%	5.2%	0.5%	20.0%	10.6%	14.8%	0.1%	0.0%
Worst	13.1%	3.2%	0.0%	14.3%	5.5%	63.8%	0.1%	0.0%

**(B) Progression‐free survival**										
**Study and rank**	**PBC**	**ATE**	**ATEPBC**	**DUR**	**IPIPBC**	**NIV**	**NIVIPI**	**NIVPBC**	**PEM**	**PEMPBC**
Best	0.0%	4.1%	6.1%	0.0%	0.7%	0.0%	35.5%	5.6%	1.0%	**47.0%**
2nd	0.0%	7.7%	21.5%	0.0%	2.2%	0.1%	22.2%	10.3%	3.5%	**32.4%**
3rd	0.0%	11.8%	**33.4%**	0.1%	5.2%	0.2%	14.4%	13.5%	8.7%	12.7%
4th	0.0%	15.2%	24.0%	0.4%	9.2%	1.0%	10.3%	17.5%	16.7%	5.7%
5th	0.4%	20.1%	10.7%	1.5%	14.6%	2.3%	7.7%	17.7%	23.2%	1.6%
6th	5.4%	17.6%	3.5%	2.7%	19.9%	4.7%	5.5%	16.3%	24.0%	0.4%
7th	26.9%	11.4%	70.0%	5.6%	20.5%	8.4%	2.6%	9.9%	14.0%	0.1%
8th	47.5%	6.3%	0.0%	9.7%	13.7%	11.1%	1.1%	4.9%	5.7%	0.0%
9th	17.8%	4.3%	0.0%	29.4%	9.6%	32.7%	0.6%	3.0%	2.6%	0.0%
Worst	1.9%	1.6%	0.0%	50.6%	4.3%	39.5%	0.1%	1.3%	0.6%	0.0%

**(C) Objective response rate**									
**Study and rank**	**PBC**	**ATEPBC**	**DUR**	**IPIPBC**	**NIV**	**NIVIPI**	**NIVPBC**	**PEM**	**PEMPBC**
Best	0.0%	0.6%	0.0%	0.0%	0.0%	24.7%	9.6%	0.1%	**65.0%**
2nd	0.0%	11.5%	0.0%	0.1%	0.0%	**33.9%**	23.8%	1.8%	28.8%
3rd	0.0%	**36.7%**	0.1%	0.4%	0.1%	20.6%	28.5%	8.1%	5.6%
4th	0.1%	41.9%	0.6%	1.9%	0.6%	12.9%	21.5%	19.7%	0.6%
5th	4.8%	8.6%	2.3%	8.9%	3.6%	6.1%	12.3%	53.3%	0.0%
6th	49.8%	0.6%	7.0%	20.1%	7.1%	1.2%	2.6%	11.7%	0.0%
7th	37.5%	0.0%	14.6%	29.1%	13.3%	0.4%	1.1%	3.9%	0.0%
8th	7.2%	0.0%	34.9%	25.6%	30.4%	0.1%	0.4%	1.3%	0.0%
Worst	0.4%	0.0%	40.5%	13.9%	44.9%	0.1%	0.2%	0.1%	0.0%

**(D) Grade 3–5 adverse events**										
**Study and rank**	**PBC**	**ATE**	**ATEPBC**	**DUR**	**IPIPBC**	**NIV**	**NIVIPI**	**NIVPBC**	**PEM**	**PEMPBC**
**Best**	0.0%	0.0%	0.3%	0.0%	49.4%	0.0%	0.0%	**50.3%**	0.0%	0.0%
**2nd**	0.0%	0.0%	8.2%	0.0%	**49.1%**	0.0%	0.0%	42.7%	0.0%	0.0%
**3rd**	0.0%	0.0%	**91.4%**	0.0%	1.5%	0.0%	0.1%	6.5%	0.0%	0.5%
**4th**	21.4%	0.0%	0.1%	0.0%	0.0%	0.0%	4.6%	0.3%	0.0%	**73.5%**
**5th**	71.9%	0.0%	0.0%	0.0%	0.0%	0.0%	5.4%	0.1%	0.0%	22.7%
**6th**	6.7%	0.1%	0.0%	0.0%	0.0%	0.0%	89.8%	0.0%	0.0%	3.4%
**7th**	0.0%	89.7%	0.0%	3.3%	0.0%	0.1%	0.1%	0.0%	6.7%	0.0%
**8th**	0.0%	8.8%	0.0%	20.4%	0.0%	1.0%	0.0%	0.0%	69.8%	0.0%
**9th**	0.0%	1.3%	0.0%	63.6%	0.0%	12.3%	0.0%	0.0%	22.8%	0.0%
**Worst**	0.0%	0.0%	0.0%	12.7%	0.0%	86.6%	0.0%	0.0%	0.7%	0.0%

*Note*: Boldface indicate the best ranking.

*Abbreviations*: ATE, atezolizumab; ATEPBC, atezolizumab and platinum‐based chemotherapy; DUR, durvalumab; IPIPBC, ipilimumab and platinum‐based chemotherapy; NIV, nivolumab; NIVIPI, nivolumab and ipilimumab; NIVPBC, nivolumab and platinum‐based chemotherapy; PBC, platinum‐based chemotherapy; PEM, pembrolizumab; PEMPBC, pembrolizumab and platinum‐based chemotherapy.

### Network meta‐analysis by PD‐L1 expression

#### PD‐L1 ≥ 50%

The OS network meta‐analysis for PD‐L1 ≥ 50% was based on 11 trials. Results from network meta‐analysis show that atezolizumab with the greatest benefit in OS over platinum‐based chemotherapy (HR 0.59, 95% CI 0.40–0.88) with the highest probability of ranking the best (47.2%; Supporting Information Table S1). All ICI treatments, except durvalumab and nivolumab, were all significantly better than platinum‐based chemotherapy in OS. However, results from network meta‐analysis show that pembrolizumab plus platinum‐based chemotherapy had the greatest benefit in PFS over platinum‐based chemotherapy (HR 0.39, 95% CI 0.27–0.56) with the highest probability of ranking the best (47.4%; Supporting Information Table S4). All ICI treatments, except atezolizumab, were significantly better than platinum‐based chemotherapy in PFS.

#### PD‐L1 1–49%

The OS network meta‐analysis for PD‐L1 1–49% was based on seven trials. Results from network meta‐analysis show that pembrolizumab plus platinum‐based chemotherapy had the greatest benefit in OS over platinum‐based chemotherapy (HR 0.60, 95% CI 0.47–0.78) with the highest probability of ranking the best (66.0%; Supporting Information Table S2). However, the PFS network meta‐analysis for PD‐L1 1–49% was based on eight trials. Results from network meta‐analysis show that pembrolizumab plus platinum‐based chemotherapy had the greatest benefit in PFS over platinum‐based chemotherapy (HR 0.51, 95% CI 0.39–0.68) with the highest probability of ranking the best (67.5%; Supporting Information Table S5).

#### PD‐L1 < 1%

The OS network meta‐analysis for PD‐L1 < 1% was based on eight trials. Results from network meta‐analysis show that pembrolizumab plus platinum‐based chemotherapy (HR 0.55, 95% CI 0.35–0.85) and nivolumab plus ipilimumab had the greatest benefit in OS over the platinum‐based chemotherapy (HR 0.53, 95% CI 0.34–0.82) with the highest probability of ranking the best (55.4%; Supporting Information Table S3). However, results from network meta‐analysis show that pembrolizumab plus platinum‐based chemotherapy had the greatest benefit in PFS over platinum‐based chemotherapy (HR 0.66, 95% CI 0.46–0.93) with the highest probability of ranking the best (29.3%; Supporting information Table S6).

### Subgroup by histology type

Fifteen trials all reported histology type including seven mixed histology types, five non‐squamous, and three squamous NSCLC patients. In the analysis of direct comparisons for OS in squamous and non‐squamous NSCLC patients all ICI treatments showed better OS than platinum‐based chemotherapy. Atezolizumab plus platinum‐based chemotherapy had the greatest benefit in OS over platinum‐based chemotherapy in squamous and non‐squamous NSCLC patients. (HR 0.38, 95% CI 0.31–0.47; HR 0.45, 95% CI 0.40–0.54, respectively) (Supporting Information Figure S3a,b). In the analysis of direct comparisons for PFS in squamous NSCLC patients, ipilimumab plus platinum‐based chemotherapy had the greatest benefit in PFS over platinum‐based chemotherapy (HR 0.42, 95% CI 0.36–0.49), followed by atezolizumab plus platinum‐based chemotherapy (HR 0.49, 95% CI 0.41–0.59) (Supporting Information Figure S3c). In the analysis of direct comparisons for PFS in non‐squamous NSCLC patients, atezolizumab plus platinum‐based chemotherapy had the greatest benefit in PFS over platinum‐based chemotherapy (HR 0.54, 95% CI 0.48–0.60) (Figure 3d).

### Publication bias and sensitivity analysis

The result of the comparison‐adjusted funnel plots did not reveal any evidence of apparent asymmetry (Supporting information Figure S2). No significant publication bias was observed. Due to one trial with small sample sizes, we have conducted this network meta‐analysis by excluding small sample sizes (Langer et al). However, a similar result found that pembrolizumab plus platinum‐based chemotherapy was still the best choice in prolonging OS and PFS for treating advanced NSCLC (HR 0.56, 95% CI 0.45–0.68; HR 0.54, 95% CI 0.39–0.75). In addition, pembrolizumab plus platinum‐based chemotherapy had higher ranked ORR than platinum‐based chemotherapy (OR 2.88, 95% CI 1.87–4.43).

## DISCUSSION

This is a network meta‐analysis discussing the efficacy and safety of ICIs as the first‐line treatment for NSCLC. Previous meta‐analyses conducted by Wang et al.^31^ have suggested that ICI‐monotherapy and ICI‐chemotherapy resulted in significantly prolonged OS and PFS compared to chemotherapy. Another previous network meta‐analysis conducted by Almutairi et al.^32^ had comparative efficacy and safety of PD‐1/PD‐L1 for previously treated advanced NSCLC. Almutairi et al. suggested that pembrolizumab and nivolumab prevailed in overall OS and ORR benefits over atezolizumab. However, subsequent studies have shown that ICIs combined with chemotherapy have a better effect on the treatment of advanced NSCLC.^15^, ^16^, ^18^, ^19^, ^20^, ^21^, ^22^, ^24^, ^25^ In 2019, a network meta‐analysis by Dafni et al.^33^ compared the efficacy of ICIs with or without chemotherapy as first‐line therapy for advanced NSCLC based on 12 phase III studies. They suggested that the combination of chemotherapy with either pembrolizumab or atezolizumab showed higher efficacy than any other therapy regimens. This network meta‐analysis has been updated and now contains 15 trials. The discrepancy of included studies between Dafni et al. and ours, three more studies were included pembrolizumab plus platinum‐based chemotherapy, ipilimumab plus platinum‐based chemotherapy, and atezolizumab. These three studies increased the total population by 1426 and provided more results for OS, PFS, ORR, and grade 3–5 TRAEs for pooling. We believe this makes our results more evidential.

Our study showed that pembrolizumab plus platinum‐based chemotherapy was the best ranking of OS, PFS, and ORR for advanced patients with NSCLC. The mechanism is not clear, but we suggest several reasons for the results. First, there are different bio‐structures and binding sites among different PD‐1/PD‐L1 inhibitors.^34^ PD‐1/PD‐L1 inhibitors bind to different PD‐1/PD‐L1 on tumors or somatic cells, which could result in different mechanisms. A study revealed that the pembrolizumab epitope region shows a much greater overlap with the PD‐L1 binding site than the epitope region of nivolumab.^35^ Second, a functional assay evaluating antibodies targeting PD‐1 inhibition in vitro revealed that pembrolizumab is a slightly more effective PD‐1 blocker than nivolumab. However, PD‐L1 antibodies are superior to PD‐1 antibodies in reverting PD‐1 signaling. A potential explanation for the lower functional half‐maximal effective concentration (EC_50_) values of PD‐L1 antibodies compared to PD‐1 antibodies is that ligands are more effectively blocked than receptors, but more work is required to address this possibility.^36^ Third, PD‐1 and PD‐L1 are expressed in different cells, for example PD‐1 is expressed on a variety of immune cells and PD‐L1 is expressed in tumor cells and antigen presenting cells.^37^ Therefore, we speculated that the number of different cells and the expression of PD‐1/PD‐L1 may affect the efficacy.

In a subgroup analysis of patients with high PD‐L1 expression (≥50%), atezolizumab had the highest probability of ranking as the best treatment for OS in first‐line treatment. However, in patients with high PD‐L1 expression (≥50%), pembrolizumab plus platinum‐based chemotherapy had the highest probability of ranking as the best treatment for PFS. Moreover, pembrolizumab plus platinum‐based chemotherapy has the highest probability of ranking for PFS regardless of the various expressions of PD‐L1. The possible reasons might attribute to this phenomenon. Each immunohistochemistry (IHC) assay was developed with a unique primary antibody (clone) against PD‐L1, namely, 28‐8 with nivolumab, 22C3 with pembrolizumab, SP263 with durvalumab, and SP142 with atezolizumab. A study demonstrated that the percentage of PD‐L1‐stained tumor cells was comparable when the 22C3, 28‐8, and SP263 assays were used, whereas the SP142 assay exhibited fewer stained tumor cells overall.^38^ Therefore, SP142 assays may underestimate the expression of PD‐L1, but in fact the PD‐L1 expression of tumor cells is very high. In the results of this study, atezolizumab seems to be useful for patients with high PD‐L1 expression (≥50%), especially when the side effects of ICI combined with chemotherapy are still higher than those of ICI alone. We also evaluated the efficacies according to histology type, and atezolizumab plus platinum‐based chemotherapy showed the greatest OS benefits over chemotherapy in both squamous and non‐squamous cancer. The result still needs to be carefully verified in the future because it was based on a few studies and direct comparisons. The performance of PD‐L1 and different histology types could be considered as the basis for choosing different PD‐L1 drugs. However, because not all studies have presented these data, this result comes from a reduced number of studies and samples, and more studies are needed to confirm this hypothesis.

This network meta‐analysis has some limitations. First, the present analysis included the different first line of treatment regimens, and this would introduce heterogeneity to the results. To address this issue, we performed detailed subgroup analyses, and similar results were found. Second, the tumor mutational burden was missed in our study, which might result in difference to our current findings. Third, unavoidable confounding factors remain in this network meta‐analysis. Because most treatments are compared indirectly, estimated effects should be used with caution.

## CONCLUSIONS

Pembrolizumab plus platinum‐based chemotherapy prevailed in rank in OS, PFS, and ORR benefit. The TRAEs of pembrolizumab plus platinum‐based chemotherapy were more than ICI monotherapy and chemotherapy. Therefore, the efficacy and safety of pembrolizumab plus platinum‐based chemotherapy should be combined in treatment decision‐making.

## CONFLICT OF INTEREST

All authors declare no conflict to declare.

## AUTHOR CONTRIBUTIONS

T.‐R.P. and T.‐W.W. wrote the first draft of the manuscript. F.‐P.T. and H.‐H.L. searched databases and extracted the data. T.‐R.P. and T.‐W.W. evaluated the risk of bias. F.‐P.T. and H.‐H.L. performed the statistical analysis. H.‐H.L. and T.‐W.W. critically revised the manuscript. All authors contributed to the final version of the manuscript.

## CONSENT FOR PUBLICATION

All authors agreed to publish.

## CODE AVAILABILITY

Software application.

## Supporting information


**Appendix S1**. Supporting Information.

## Data Availability

All data, models, and code generated or used during the study appear in the submitted article.
