# Supplementary material for: Immune checkpoint inhibitors for first‐line treatment of advanced non‐small‐cell lung cancer: A systematic review and network meta‐analysis
Source: Thorac Cancer. 2021 Sep 20;12(21):2873–85. doi: 10.1111/1759-7714.14148 (PMC8563153; doi:10.1111/1759-7714.14148)
Supplement: Supplementary file 1 — Appendix S1. Supporting Information. [file TCA-12-2873-s001.docx]

**
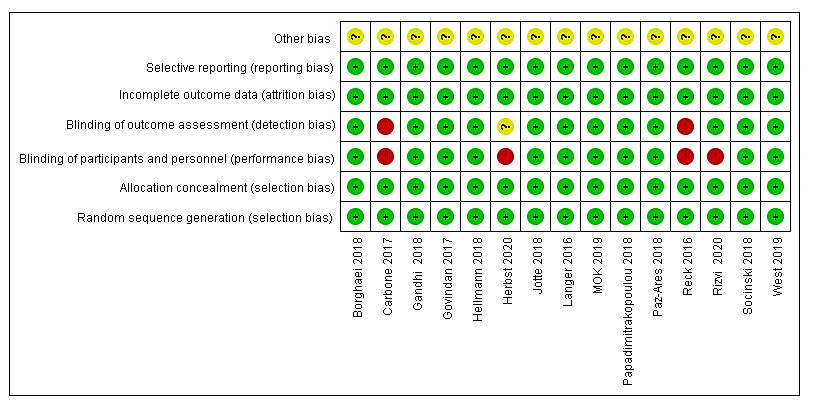
**

Supplementary Figure 1. Assessment of methodological quality of included trials


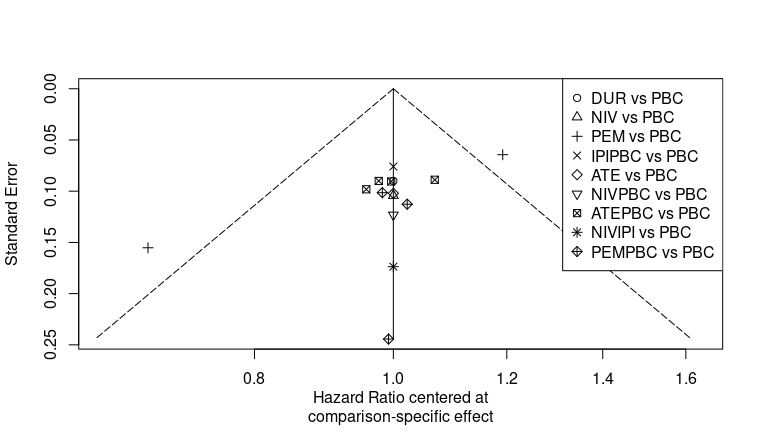


Supplementary Figure 2. Comparison-adjusted funnel plot for the selected studies. Abbreviation: ATE, Atezolizumab; ATEPBC, Atezolizumab & Platinum-based chemotherapy; DUR, Durvalumab; IPIPBC, Ipilimumab & Platinum-based chemotherapy; NIV, Nivolumab; NIVIPI, Nivolumab & Ipilimumab; NIVPBC, Nivolumab & Platinum-based chemotherapy; PEM, Pembrolizumab; PEMPBC, Pembrolizumab & Platinum-based chemotherapy; PBC, Platinum-based chemotherapy.


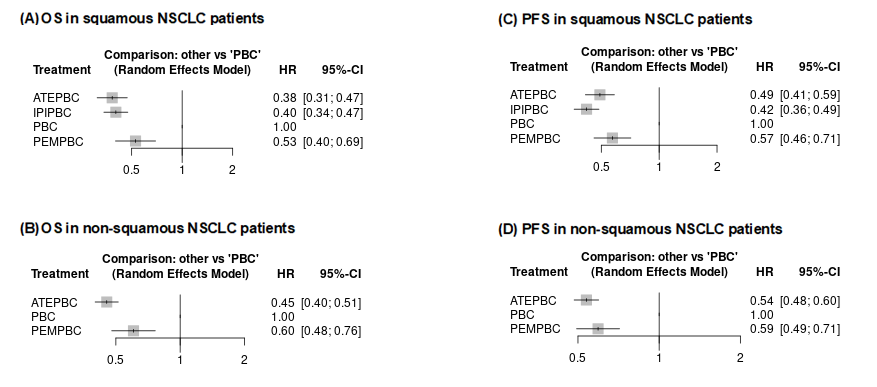


Supplementary Figure 3. Network Meta-Analysis in histology type subgroups. (A) Overall Survival in squamous NSCLC patients; (B) Overall Survival in non- squamous NSCLC patients; (C) Progression-Free Survival in squamous NSCLC patients; (D) Progression-Free Survival in non- squamous NSCLC patients. Abbreviation: ATE, Atezolizumab; ATEPBC, Atezolizumab & Platinum-based chemotherapy; DUR, Durvalumab; IPIPBC, Ipilimumab & Platinum-based chemotherapy; NIV, Nivolumab; NIVIPI, Nivolumab & Ipilimumab; NIVPBC, Nivolumab & Platinum-based chemotherapy; PEM, Pembrolizumab; PEMPBC, Pembrolizumab & Platinum-based chemotherapy; PBC, Platinum-based chemotherapy.

Supplementary Table 1. Rank probability of treatment for OS subgroup of the PD-L1 ≥ 50%.

| **Study and rank** | **PBC** | **ATE** | **ATEPBC** | **DUR** | **NIV** | **NIVIPI** | **PEM** | **PEMPBC** |
| --- | --- | --- | --- | --- | --- | --- | --- | --- |
| Best | 0.0% | **47.2%** | 21.2% | 4.4% | 0.6% | 6.8% | 6.6% | 13.2% |
| 2nd | 0.0% | 17.5% | **22.4%** | 8.0% | 1.5% | 13.9% | 20.1% | 16.6% |
| 3rd | 0.0% | 10.4% | 15.5% | 9.5% | 2.5% | 17.7% | **27.3%** | 17.1% |
| 4th | 0.0% | 8.3% | 13.4% | 13.4% | 4.4% | 19.2% | 24.8% | 16.5% |
| 5th | 0.1% | 8.0% | 13.0% | 17.5% | 8.4% | 20.8% | 14.6% | 17.7% |
| 6th | 1.9% | 5.9% | 10.2% | 27.7% | 18.1% | 16.5% | 5.9% | 13.8% |
| 7th | 30.8% | 2.3% | 3.9% | 15.4% | 37.2% | 5.0% | 0.8% | 4.6% |
| Worst | 67.2% | 0.3% | 0.4% | 4.2% | 27.2% | 0.1% | 0.0% | 0.5% |
| Abbreviation: ATE, Atezolizumab; ATEPBC, Atezolizumab & Platinum-based chemotherapy; DUR, Durvalumab; IPIPBC, Ipilimumab & Platinum-based chemotherapy; NIV, Nivolumab; NIVIPI, Nivolumab & Ipilimumab; NIVPBC, Nivolumab & Platinum-based chemotherapy; PEM, Pembrolizumab; PEMPBC, Pembrolizumab & Platinum-based chemotherapy; PBC, Platinum-based chemotherapy. | | | | | | | | |

Supplementary Table 2. Rank probability of treatment for OS subgroup of the PD-L1 1%-49%.

| **Study and rank** | **PBC** | **ATE** | **ATEPBC** | **PEM** | **PEMPBC** |
| --- | --- | --- | --- | --- | --- |
| **Best** | 0.0% | 33.6% | 0.0% | 0.4% | **66.0%** |
| **2nd** | 4.1% | 10.8% | 12.4% | **38.9%** | 33.7% |
| **3rd** | 29.7% | 2.8% | 24.8% | **42.5%** | 0.2% |
| **4th** | 45.8% | 2.5% | 38.1% | 13.5% | 0.0% |
| **Worst** | 20.4% | 50.3% | 24.7% | 4.6% | 0.0% |
| Abbreviation: ATE, Atezolizumab; ATEPBC, Atezolizumab & Platinum-based chemotherapy; DUR, Durvalumab; IPIPBC, Ipilimumab & Platinum-based chemotherapy; NIV, Nivolumab; NIVIPI, Nivolumab & Ipilimumab; NIVPBC, Nivolumab & Platinum-based chemotherapy; PEM, Pembrolizumab; PEMPBC, Pembrolizumab & Platinum-based chemotherapy; PBC, Platinum-based chemotherapy. | | | | | |

Supplementary Table 3. Rank probability of treatment for OS subgroup of the PD-L1 <1%.

| **Study and rank** | **PBC** | **ATEPBC** | **DUR** | **NIVIPI** | **NIVPBC** | **PEMPBC** |
| --- | --- | --- | --- | --- | --- | --- |
| **Best** | 0.0% | 1.6% | 0.0% | **55.4%** | 5.1% | 37.8% |
| **2nd** | 0.0% | 9.3% | 0.2% | 31.5% | 16.3% | **42.7%** |
| **3rd** | 0.0% | **38.1%** | 0.7% | 9.8% | 37.0% | 14.3% |
| **4th** | 4.4% | 47.7% | 3.5% | 3.2% | 36.2% | 5.0% |
| **5th** | 78.8% | 3.0% | 13.6% | 0.1% | 4.4% | 0.2% |
| **Worst** | 16.8% | 0.3% | 82.0% | 0.0% | 1.0% | 0.0% |
| Abbreviation: ATE, Atezolizumab; ATEPBC, Atezolizumab & Platinum-based chemotherapy; DUR, Durvalumab; IPIPBC, Ipilimumab & Platinum-based chemotherapy; NIV, Nivolumab; NIVIPI, Nivolumab & Ipilimumab; NIVPBC, Nivolumab & Platinum-based chemotherapy; PEM, Pembrolizumab; PEMPBC, Pembrolizumab & Platinum-based chemotherapy; PBC, Platinum-based chemotherapy. | | | | | | |

Supplementary Table 4. Rank probability of treatment for PFS subgroup of the PD-L1 ≥ 50%.

| **Study and rank** | **PBC** | **ATE** | **ATEPBC** | **IPIPBC** | **NIV** | **NIVIPI** | **PEM** | **PEMPBC** |
| --- | --- | --- | --- | --- | --- | --- | --- | --- |
| **Best** | 0.0% | 2.6% | 35.3% | 12.7% | 0.0% | 1.9% | 0.1% | **47.4%** |
| **2nd** | 0.0% | 6.5% | **36.6%** | 17.5% | 0.0% | 5.6% | 1.1% | 32.6% |
| **3rd** | 0.0% | 14.6% | 19.4% | **27.9%** | 0.3% | 15.4% | 7.7% | 14.6% |
| **4th** | 0.0% | 22.6% | 6.3% | 18.9% | 1.2% | 25.4% | 21.5% | 4.1% |
| **5th** | 0.1% | 24.3% | 2.0% | 11.9% | 3.1% | 25.0% | 32.6% | 1.1% |
| **6th** | 3.4% | 22.4% | 0.4% | 9.1% | 9.6% | 22.2% | 32.7% | 0.2% |
| **7th** | 58.6% | 5.4% | 0.0% | 1.5% | 27.0% | 3.5% | 3.9% | 0.0% |
| **Worst** | 37.9% | 1.6% | 0.0% | 0.5% | 58.8% | 1.0% | 0.2% | 0.0% |
| Abbreviation: ATE, Atezolizumab; ATEPBC, Atezolizumab & Platinum-based chemotherapy; DUR, Durvalumab; IPIPBC, Ipilimumab & Platinum-based chemotherapy; NIV, Nivolumab; NIVIPI, Nivolumab & Ipilimumab; NIVPBC, Nivolumab & Platinum-based chemotherapy; PEM, Pembrolizumab; PEMPBC, Pembrolizumab & Platinum-based chemotherapy; PBC, Platinum-based chemotherapy. | | | | | | | | |

Supplementary Table 5. Rank probability of treatment for PFS subgroup of the PD-L1 1%-49%.

| **Study and rank** | **PBC** | **ATE** | **ATEPBC** | **IPIPBC** | **PEM** | **PEMPBC** |
| --- | --- | --- | --- | --- | --- | --- |
| **Best** | 0.0% | 0.3% | 7.9% | 24.4% | 0.0% | **67.5%** |
| **2nd** | 0.0% | 2.0% | **38.5%** | 33.9% | 0.0% | 25.6% |
| **3rd** | 0.4% | 9.6% | **49.3%** | 34.0% | 0.1% | 6.6% |
| **4th** | 27.9% | 58.3% | 4.3% | 6.8% | 2.3% | 0.3% |
| **5th** | 67.4% | 23.8% | 0.0% | 0.7% | 8.1% | 0.0% |
| **Worst** | 4.3% | 6.0% | 0.0% | 0.2% | 89.5% | 0.0% |
| Abbreviation: ATE, Atezolizumab; ATEPBC, Atezolizumab & Platinum-based chemotherapy; DUR, Durvalumab; IPIPBC, Ipilimumab & Platinum-based chemotherapy; NIV, Nivolumab; NIVIPI, Nivolumab & Ipilimumab; NIVPBC, Nivolumab & Platinum-based chemotherapy; PEM, Pembrolizumab; PEMPBC, Pembrolizumab & Platinum-based chemotherapy; PBC, Platinum-based chemotherapy. | | | | | | |

Supplementary Table 6. Rank probability of treatment for PFS subgroup of the PD-L1 <1%.

| **Study and rank** | **PBC** | **ATE** | **ATEPBC** | **IPIPBC** | **NIVIPI** | **PEMPBC** |
| --- | --- | --- | --- | --- | --- | --- |
| **Best** | 0.0% | 18.9% | 17.7% | 18.8% | 15.3% | **29.3%** |
| **2nd** | 0.0% | 16.3% | **26.4%** | 16.9% | 14.9% | 25.5% |
| **3rd** | 0.2% | 17.3% | **26.8%** | 18.5% | 16.9% | 20.4% |
| **4th** | 2.6% | 20.0% | 19.8% | 19.9% | 21.7% | 16.0% |
| **5th** | 23.2% | 19.6% | 9.0% | 18.7% | 21.2% | 8.3% |
| **Worst** | 74.0% | 7.9% | 0.4% | 7.2% | 10.0% | 0.6% |
| Abbreviation: ATE, Atezolizumab; ATEPBC, Atezolizumab & Platinum-based chemotherapy; DUR, Durvalumab; IPIPBC, Ipilimumab & Platinum-based chemotherapy; NIV, Nivolumab; NIVIPI, Nivolumab & Ipilimumab; NIVPBC, Nivolumab & Platinum-based chemotherapy; PEM, Pembrolizumab; PEMPBC, Pembrolizumab & Platinum-based chemotherapy; PBC, Platinum-based chemotherapy. | | | | | | |
